# Cutting through the clones: genomic strategies for core collection development in moso bamboo

**DOI:** 10.1186/s12864-026-12548-7

**Published:** 2026-01-20

**Authors:** Rui Gu, Songpo Wei, Shaohui Fan, Martha Rendón-Anaya, Guanglu Liu

**Affiliations:** 1 International Center for Bamboo and Rattan, Beijing, China; 2 Key Laboratory of National Forestry and Grassland Administration for Bamboo & Rattan Science and Technology, Beijing, China; 3 Department of Plant Biology, Linnean Centre for Plant Biology, Swedish University of Agricultural Sciences, Uppsala, Sweden

**Keywords:** Core collection, Genetic structure, Moso bamboo, Genetic diversity, Phenotypic variation

## Abstract

**Background:**

Conserving genetic diversity is crucial for effective germplasm use and crop improvement. Developing core collections with minimal redundancy and maximum diversity requires a clear understanding of population structure. However, the nationwide population structure of moso bamboo (*Phyllostachys edulis*) remains poorly characterized, creating a major gap for developing representative, non-redundant core collections.

**Results:**

Using whole-genome resequencing data from 432 moso bamboo accessions covering a broad geographic range across the distribution of the species in China, we investigated the population genetic structure and diversity patterns. Principal component analysis and phylogenetic tree analyses identified three distinct genetic clusters together with a hybrid group. To identify the optimal strategy for core collection development, we evaluated two stratification schemes, seven sampling strategies, and five sampling intensities. Across 70 candidate cores, stratified sampling combined with expected heterozygosity optimization at 20% intensity (S-HE20) maximized genetic diversity (He = 0.3665; PIC = 0.2904; I = 0.5302) and captured broad phenotypic variation (CR = 82.32%; MD = 0%), yielding an 84-accession core spanning 15 geographic regions.

**Conclusions:**

This study revealed the population genetic structure of moso bamboo and identified the S-HE20 strategy as optimal for core collection construction. The resulting core collection offers a representative and genetically diverse resource for future gene discovery and molecular breeding efforts in moso bamboo.

**Supplementary Information:**

The online version contains supplementary material available at 10.1186/s12864-026-12548-7.

## Background

Moso bamboo (*Phyllostachys edulis* (Carrière) J. Houz.) is a fast-growing and highly valuable non-timber forest species, widely utilized in construction, furniture, papermaking, and handicrafts due to its excellent mechanical properties [1]. It accounts for approximately 70% of China’s total bamboo cultivation area, covering around 5.28 million hectares [2]. Moso bamboo plays a crucial role in the national bamboo industry, which was valued at over USD 21 billion in 2023 [3]. Its wide distribution and predominantly clonal reproduction have led to extensive cultivation but also increased vulnerability to climate change and habitat fragmentation. Despite its economic significance, current germplasm resources are large but poorly characterized, and several key gaps limit their effective use: unclear population structure, lack of well-defined core collections, and a narrow genetic base in breeding programs. These limitations constrain the sustainable growth of the bamboo industry.

The concept of a core collection—a representative subset of accessions that retains maximum genetic diversity with minimal redundancy—was first proposed by Frankel [4]. Such collections provide efficient entry points for genetic studies and breeding, and have been successfully applied in QTL mapping, genetic mapping, and gene discovery in crops including soybean [5, 6], maize [7], Chinese cabbage [8], and jujube [9]. Developing a core collection generally comprises four main steps: (1) data acquisition (molecular and/or phenotypic), (2) accession grouping based on genetic or ecological criteria, (3) strategic sampling, and (4) evaluation of diversity metrics such as allele richness or phenotypic variance [10, 11]. Despite their utility in crop breeding, core collections remain underutilized in forest trees—especially clonal species like bamboo. Existing efforts face three key limitations: (1) most rely solely on either phenotypic traits or molecular markers, which only partially capture the underlying genetic variation [12, 13]; (2) non-stratified random sampling is still commonly used, potentially overlooking population substructure [14, 15]; (3) traditional algorithms such as the M-strategy, PowerCore, and GenoCore often optimize a single objective (e.g., maximizing allelic richness), lacking flexibility to balance multiple goals such as genetic distance, trait variation, and rare allele retention [16–18]. Recently, Core Hunter v3 has been widely adopted as an effective tool for multi-objective core collection optimization, offering flexible metrics and diverse sampling strategies [19]. It incorporates both distance-based metrics—such as entry-to-nearest-entry (E-NE) and accession-to-nearest-entry (A-NE)—and diversity indices such as Shannon’s diversity index (SH) and expected heterozygosity (HE), enabling simultaneous optimization of representativeness and diversity. The use of different genetic distance measures, including Modified Rogers’ Distance (MR) and Cavalli–Sforza and Edwards’ distance (CE), further refines sensitivity to allele frequency patterns and rare variants.

In this study, we conducted the first comprehensive effort to construct a core collection for moso bamboo. Using whole-genome resequencing data from 432 accessions representing a broad geographic range of the species’ distribution in China, we analyzed population structure and genetic diversity. We then evaluated multiple core collection strategies using Core Hunter v3, testing: (1) stratified versus non-stratified sampling, (2) a range of selection algorithms, and (3) different sampling proportions. Our work provides a scientific foundation for cost-effective conservation and enhanced utilization of moso bamboo genetic resources, while offering methodological guidance for core collection development in other clonally propagated forest species.

## Results

### SNP discovery

A total of 19.71 Tb of sequencing data were generated from 432 moso bamboo individuals, at a mean sequencing depth of 20.93 × (Additional file 1: Table S1). Sequencing reads were mapped to the chromosome-scale reference genome of moso bamboo, yielding an average mapping rate of 95.41% and a mean genome coverage of 92.06% (Additional file 1: Table S1). After quality control and variant filtering, 58.43 million high-quality SNPs were identified (Additional file 2: Table S2), corresponding to an average genome-wide SNP density of one SNP per 30 bp. Among these variants, 68.62% were located in intergenic regions, 5.79% in introns, and 1.33% in coding sequences (Additional file 3: Table S3). The transition/transversion ratio was 3.41, and the nonsynonymous/synonymous ratio for all biallelic coding SNPs was 1.43 (Additional file 3: Table S3).

### Population genetic structure

**Fig. 1:**
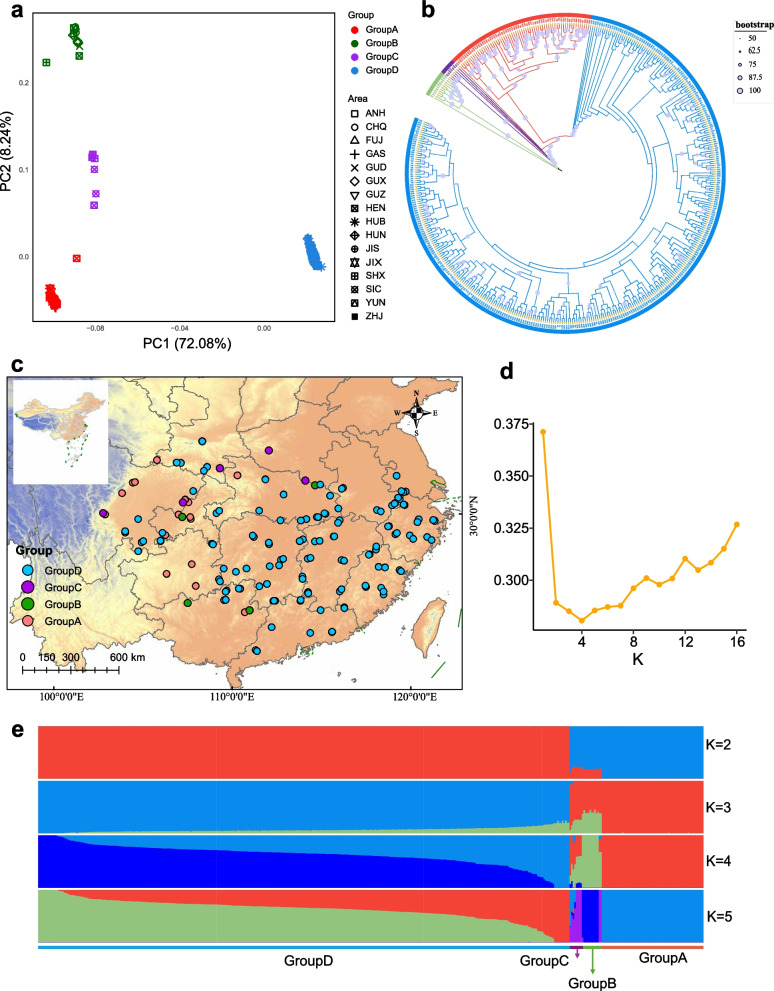
Population genomic analyses of moso bamboo. **a** Principal component analysis (PCA) performed in PLINK v1.90 using 329,747 LD-pruned SNPs (MAF ≥ 0.05). Colors represent inferred genetic clusters and shapes indicate geographic regions. **b** Maximum-likelihood phylogenetic tree reconstructed using IQ-TREE v2 with the MFP model and 1,000 bootstrap replicates; the outer ring indicates the four major genetic groups. **c** Geographical distribution of all 432 accessions across China, colored by inferred genetic cluster. **d** Cross-validation error values for K = 2–16 from ADMIXTURE v1.3.0, showing K = 4 as the optimal clustering. **e** Population structure inferred by ADMIXTURE v1.3.0 at K = 2–5 using the 329,747 LD-pruned SNPs; each vertical bar represents one accession

Principal component analysis (PCA) identified four distinct genetic clusters (Groups A–D). The first principal component (PC1, explaining 72.08% of total variance) clearly separated Group D from the other groups, while the second component (PC2, explaining 8.24%) distinguished Group A from Groups B and C (Fig. 1a). The unusually high proportion of variance explained by PC1 reflects the high clonal redundancy and pronounced long-term lineage divergence characteristic of moso bamboo. A similar pattern of genetic divergence was observed in the maximum likelihood (ML) phylogenetic tree, supporting the PCA results (Fig. 1b). ADMIXTURE analysis further confirmed four genetic clusters (K = 4) as the optimal grouping, based on the lowest cross-validation error (Fig. 1d). At K = 2, individuals were broadly separated into western (Group A) and eastern (Group D) lineages. At K = 3, a third cluster appeared, largely derived from the western lineage. At the optimal K = 4, a distinct ancestral component emerged almost exclusively within Group D, where it constituted the dominant proportion of ancestry. This Group D–specific component, represented by the deep-blue segment in Fig. 1e, corresponds to accessions primarily distributed in the southeastern coastal region and reveals substructure not captured by PCA or the ML phylogeny. Notably, individuals from Group C (hybrid group, 7 individuals) exhibited strong ancestral admixture from multiple clusters, suggesting genetic complexity and a possible hybrid origin. Geographically, Group D was primarily distributed in southeastern coastal regions, Group A in the southwestern highlands (transition zone between the Yunnan–Guizhou Plateau and western Sichuan Mountains), and Groups B and C in the north and northwest, including northern Guizhou, central to northern Sichuan, and southern Shanxi (Fig. 1c). Despite this, the overall genetic structure lacked a clear phylogeographic pattern, possibly due to China’s complex topography limiting historical gene flow, and recent human-mediated clonal propagation and translocation, which likely disrupted the original population structure.

### Genetic diversity

To assess genome-wide patterns of genetic diversity and differentiation, individuals from the hybrid group were excluded from the analysis. Among the remaining three genetic groups, Group D exhibited the highest levels of genetic diversity, as indicated by the highest observed heterozygosity (Ho = 0.4967), expected heterozygosity (He = 0.3216), polymorphism information content (PIC = 0.2563) and Shannon’s diversity index (I = 0.4789) (Table 1). In contrast, Group B showed the lowest diversity, with the lowest Ho (0.3281), He (0.2942), PIC (0.2339) and I (0.4013) (Table 1). Across all groups, Ho exceeded He, reflecting an excess of heterozygosity. This is consistent with the predominantly clonal reproduction of moso bamboo, which preserves heterozygous genotypes, with historical admixture and genome duplication further reinforcing this pattern.

**Table 1:** Genetic diversity parameters of the three groups

Group	Ho	He	PIC	I	J′
GroupA	0.4023	0.2975	0.2363	0.4271	0.7288
GroupB	0.3281	0.2942	0.2339	0.4013	0.7471
GroupD	0.4967	0.3216	0.2563	0.4789	0.7346

*Ho* Observed heterozygosity, *He* Expected heterozygosity, *PIC* Polymorphism information content, *I* Shannon’s diversity index, *J′* Genetic evenness

### Core collection optimization, development and evaluation

To assess the effectiveness of core collection selection methods, we compared seven optimization strategies under both unstratified and stratified sampling approaches across five sampling proportions (10%, 15%, 20%, 25%, and 30%). Genetic diversity indices, including PIC, He, Ho, I, and J′, were used as evaluation metrics. Hybrid accessions were excluded from the stratified sampling analyses because they show admixed ancestry and do not form a distinct population cluster. In addition, their low number (*n* = 7) makes proportional sample allocation across groups infeasible at multiple sampling intensities, which would compromise the assumptions of stratified sampling. In contrast, these accessions were retained in the unstratified framework, where sampling is performed across the full dataset without relying on predefined group structure.

Overall, stratified sampling consistently outperformed unstratified sampling in capturing genetic diversity. For instance, at a 10% sampling intensity using the HE method, stratified sampling yielded a higher Ho (0.5160 vs. 0.4632) compared to unstratified sampling (Table 2). Notably, under the EN-MR method, He dropped from 0.3479 (stratified) to 0.2038 (unstratified), highlighting stratified sampling's advantage in preserving within-group variation and avoiding redundancy from dominant genotypes (Table 2). Under the stratified sampling strategy, we further assessed the performance of different sampling methods across varying sampling proportions. Among all stratified sampling combinations, the HE method at 10% and 20% sampling proportions consistently showed the highest genetic diversity and evenness. Specifically, at 10%, the HE method produced the highest Ho (0.5160) and J′ (0.7658), while at 20%, it achieved the highest values of He (0.3665), PIC (0.2904), and I (0.5302), reflecting effective representation of genetic variability. Based on these results, the HE method under stratified sampling, particularly at 10% and 20% sampling proportions, was identified as the most effective strategy. Therefore, the S-HE10 (Stratified sampling with HE method at 10% proportion) and S-HE20 (Stratified sampling with HE method at 20% proportion) subsets were selected as candidate core collections.

**Table 2:** Genetic diversity parameters under different sampling strategies

Sampling ratio %	Sampling methods	Stratified sampling					Unstratified sampling				
		Ho	He	PIC	I	J′	Ho	He	PIC	I	J′
10%	AN_CE	0.4852	0.3509	0.2795	0.5157	0.7444	0.4008	0.2826	0.1542	0.5127	0.7412
	AN_MR	0.4853	0.3509	0.2795	0.5158	0.7445	0.4018	0.2830	0.1533	0.5130	0.7416
	EN_CE	0.4757	0.3477	0.2771	0.5103	0.7367	0.3808	0.2039	0.2657	0.4691	0.6920
	EN_MR	0.4762	0.3479	0.2772	0.5105	0.7370	0.3807	0.2038	0.2653	0.4691	0.6918
	ENMR0.5_SH0.5	0.4996	0.3572	0.2834	0.5228	0.7553	0.4224	0.3269	0.2299	0.5329	0.7692
	HE	0.5160	0.3616	0.2862	0.5294	0.7658	0.4632	0.3612	0.3043	0.5505	0.7949
	SH	0.5151	0.3613	0.2861	0.5292	0.7650	0.4632	0.3612	0.2725	0.5505	0.7949
15%	AN_CE	0.4823	0.3618	0.2876	0.5231	0.7546	0.4005	0.2570	0.1057	0.4912	0.7104
	AN_MR	0.4818	0.3618	0.2876	0.5228	0.7543	0.4006	0.2619	0.1163	0.4958	0.7165
	EN_CE	0.4758	0.3598	0.2860	0.5195	0.7495	0.4032	0.2855	0.1653	0.5066	0.7314
	EN_MR	0.4729	0.3586	0.2852	0.5177	0.7469	0.4070	0.2965	0.1862	0.5116	0.7385
	ENMR0.5_SH0.5	0.4919	0.3659	0.2900	0.5276	0.7614	0.4477	0.3563	0.2720	0.5408	0.7803
	HE	0.5042	0.3690	0.2921	0.5330	0.7691	0.4649	0.3654	0.2807	0.5480	0.7907
	SH	0.5032	0.3685	0.2918	0.5324	0.7683	0.4649	0.3654	0.2807	0.5480	0.7907
20%	AN_CE	0.4867	0.3614	0.2872	0.5228	0.7542	0.4018	0.2586	0.1135	0.4888	0.7060
	AN_MR	0.4866	0.3613	0.2872	0.5227	0.7541	0.4022	0.2596	0.1158	0.4888	0.7061
	EN_CE	0.4793	0.3587	0.2853	0.5187	0.7483	0.4271	0.3249	0.2356	0.5202	0.7505
	EN_MR	0.4785	0.3584	0.2851	0.5180	0.7473	0.4197	0.3183	0.2247	0.5194	0.7494
	ENMR0.5_SH0.5	0.4944	0.3648	0.2894	0.5267	0.7598	0.4553	0.3621	0.2804	0.5406	0.7800
	HE	0.5036	0.3665	0.2904	0.5302	0.7650	0.4642	0.3653	0.2816	0.5468	0.7890
	SH	0.5003	0.3648	0.2894	0.5284	0.7624	0.4642	0.3653	0.2816	0.5468	0.7890
25%	AN_CE	0.4844	0.3621	0.2874	0.5230	0.7545	0.4207	0.3082	0.2092	0.5149	0.7429
	AN_MR	0.4845	0.3621	0.2874	0.5230	0.7546	0.4207	0.3082	0.2092	0.5149	0.7429
	EN_CE	0.4805	0.3605	0.2862	0.5207	0.7512	0.4361	0.3455	0.2644	0.5285	0.7624
	EN_MR	0.4810	0.3607	0.2864	0.5209	0.7515	0.4363	0.3441	0.2626	0.5286	0.7626
	ENMR0.5_SH0.5	0.4940	0.3656	0.2896	0.5276	0.7612	0.4625	0.3661	0.2862	0.5402	0.7794
	HE	0.5002	0.3665	0.2902	0.5298	0.7644	0.4645	0.3661	0.2843	0.5447	0.7859
	SH	0.5001	0.3665	0.2903	0.5299	0.7644	0.4645	0.3661	0.2843	0.5447	0.7859
30%	AN_CE	0.4851	0.3609	0.2872	0.5228	0.7543	0.4361	0.3385	0.2545	0.5275	0.7611
	AN_MR	0.4848	0.3608	0.2871	0.5227	0.7541	0.4361	0.3385	0.2546	0.5276	0.7611
	EN_CE	0.4824	0.3595	0.2861	0.5208	0.7514	0.4464	0.3556	0.2768	0.5308	0.7659
	EN_MR	0.4814	0.3593	0.2859	0.5204	0.7508	0.4504	0.3559	0.2781	0.5293	0.7636
	ENMR0.5_SH0.5	0.4916	0.3632	0.2886	0.5259	0.7587	0.4621	0.3646	0.2850	0.5390	0.7776
	HE	0.4981	0.3643	0.2893	0.5283	0.7622	0.4639	0.3662	0.2853	0.5425	0.7827
	SH	0.4980	0.3642	0.2893	0.5283	0.7622	0.4639	0.3662	0.2853	0.5425	0.7827

*Ho* Observed heterozygosity, *He* Expected heterozygosity, *PIC* Polymorphism information content, *I* Shannon’s diversity index, *J′* Genetic evenness

To evaluate the phenotypic representativeness of the selected core collections (S-HE10 and S-HE20), we assessed four statistical metrics across 15 phenotypic traits. Both collections exhibited an MD of 0, indicating no significant difference in mean trait values compared to the entire collection and satisfying the criterion of MD < 20% (Table 3). S-HE20 achieved a CR of 82.32%, surpassing the 80% threshold, whereas S-HE10 reached 72.82%, falling slightly below (Table 3). In addition, S-HE20 demonstrated the best performance in variance-based metrics (VR = 101.72%, VD = 80.33%). Collectively, these findings validate S-HE20 as the optimal core collection, with comprehensive phenotypic representativeness and full compliance with selection standards. The final core collection comprises 84 accessions distributed across 15 geographic regions, including 13 from Group A, 2 from Group B, and 69 from Group D (Fig. 1, Additional file 4: Table S4).

**Table 3:** Evaluation parameters of core collections subset

Sampling methods	Sampling Ratio%	MD%	VD%	CR%	VR%
HE	10%	0	13.33	72.82	95.98
	20%	0	80.33	82.32	101.72

*MD%* Mean difference percentage, *CR%* coincidence rate of range, *VD%* variance difference percentage, *VR%* coefficient of variation change

### Validation of core collections

**Fig. 2:**
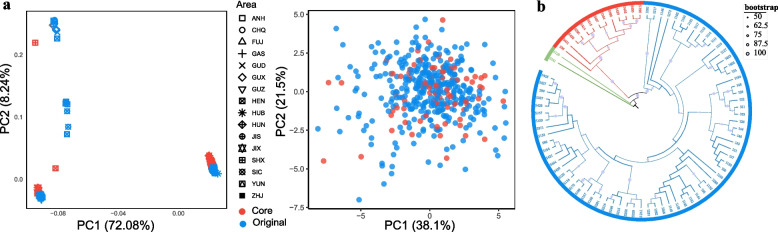
Evaluation of the representativeness of the core collection. **a** PCA plots showing the distribution of core and original collections, with the left plot based on genotypic data (329,747 LD-pruned SNPs) and the right plot based on 15 phenotypic data. Different colored dots represent core and original accessions, respectively. **b** Phylogenetic tree of the core collection

**Fig. 3:**
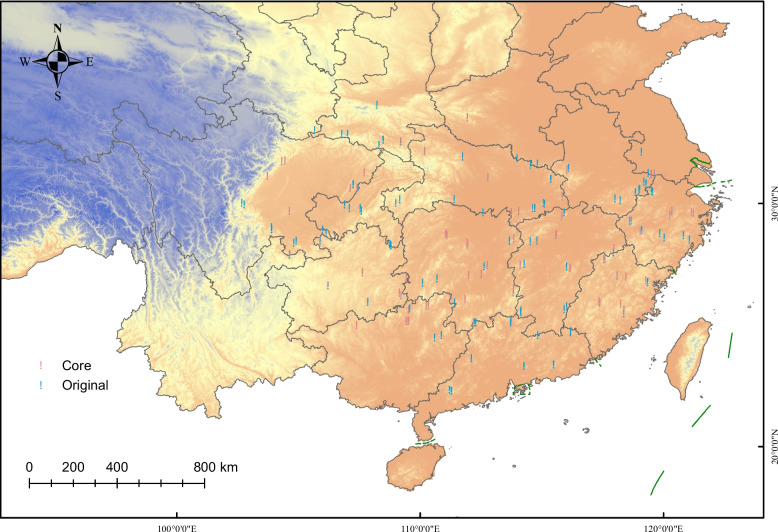
Geographical distribution of the original collection (blue, *n* = 432) and the selected core collection (red, *n* = 84) across the range of moso bamboo in China. Blue dots represent all sampled accessions, whereas red dots indicate those included in the final core collection

The core collection exhibited a higher Ho, while other genetic indices (He, PIC, I, J′) and all phenotypic indicators (maximum, minimum, mean, CV) showed no significant differences compared to the original collection, indicating effective preservation of genetic and phenotypic diversity (Table 4, Additional file 5: Table S5). In the genomic PCA, the core collection preserves the underlying genetic structure of the full dataset. The phenotypic PCA, meanwhile, shows reduced point density, reflecting the removal of phenotypically redundant accessions: the accession number decreased from 432 to 84 (an 80.6% reduction) while maintaining the overall distribution of the original collection, as indicated by a high coincidence rate (CR = 82.32%) and a mean difference of zero (MD = 0%) (Fig. 2). For phenotypic data, the eigenvalues, contribution rates, cumulative contribution rates, and major contributing traits were highly consistent between the core and original collections. The cumulative contribution rates of the first three principal components were 70.90% and 67.61%, respectively, indicating that the construction of the core collection not only preserved the main genetic information but also effectively eliminated genetic redundancy, thereby improving the cumulative contribution rate (Table 5). Notably, the core collection also retained accessions located at the edges of the phenotypic PCA space, demonstrating that it preserved not only common types but also unique or extreme phenotypes present in the full collection (Fig. 2). Core collection accessions were distributed across different clades in the phylogenetic tree (Fig. 2). These findings collectively suggest that the constructed core collection provides a comprehensive and representative sampling of the genetic diversity present in the entire population and geographic range, as depicted in Fig. 3.

**Table 4:** Genetic diversity parameters of original and core collections

Collection	Ho	He	PIC	I	J'
Original	0.4754^b^	0.3552^a^	0.2829^a^	0.5160^a^	0.7444^a^
Core	0.5036^a^	0.3664^a^	0.2904^a^	0.5302^a^	0.7650^a^

*Ho* Observed heterozygosity, *He* Expected heterozygosity, *PIC* Polymorphism information content, *I* Shannon’s diversity index, *J'* Genetic evenness. The same letter denotes that genetic diversity parameters do not differ significantly between the original and core collections

**Table 5:** Principal component analysis of phenotypic traits for original and core collections

Component	Original collection				Core collection			
	Eigen value	Contribution percentage %	Cumulative contribution percentage %	The top three contributing traits	Eigen value	Contribution percentage %	Cumulative contribution percentage %	The top three contributing traits
PC1	2.39	38.10	38.10	DBH,CCD,BD	2.50	41.55	41.55	DBH,CCD,BD
PC2	1.80	21.53	59.63	LA,LW,LL	1.73	20.02	61.56	LA,LL,LW
PC3	1.09	7.97	67.61	CW,HFB,IL	1.18	9.33	70.90	HFB,IL,CW

*DBH* Diameter at breast height, *CCD* Culm cavity diameter, *BD* Basal diameter, *LA* Leaf area, *LW* Leaf width, *LL* Leaf length, *CW* Crown width, *HFB* Height to the first branch, *IL* Internode length

## Discussion

Although previous studies have investigated the genetic diversity of moso bamboo, most were limited to major production regions. In this study, we performed an extensive analysis using whole-genome resequencing data from 432 accessions collected across the broad distribution of the species in China. We found that the moso bamboo germplasm comprises three distinct genetic clusters along with a hybrid group (Fig. 1). Among them, group D is the most widely distributed across the territory and exhibits the highest degree of genetic redundancy. We observed high levels of heterozygosity, a pattern that is consistent with previous findings in clonally propagated perennial species [20, 21]. The pervasive heterozygosity observed in moso bamboo is most likely driven by its long-term clonal propagation, which suppresses meiotic recombination and thus preserves ancestral heterozygous genotypes across generations. In addition, somatic mutations accumulated over time within and among clonal lineages contribute further heterozygous variants [22].

Recent progress in high-throughput sequencing technologies has greatly accelerated the management and utilization of plant germplasm resources by enabling precise and efficient detection of genetic variation at the genome-wide scale. However, with the continued expansion of germplasm repositories, challenges have emerged regarding accurate identification, efficient conservation, and the efficient utilization of germplasm. To address these issues, Frankel [4] proposed the concept of a “core collection”—a representative subset of accessions that retains the greatest possible genetic diversity with the least redundancy—thereby enhancing the efficiency of germplasm use. Core collections have since become a vital tool in genetic improvement and diversity conservation, with their “representativeness” heavily dependent on well-designed sampling strategies and algorithms. Previous studies have evaluated various core collection construction methods. Thachuk et al. [23] compared algorithms such as the D-method, MSTRAT, PowerCore, and Core Hunter, concluding that Core Hunter outperformed others in preserving both overall genetic diversity and rare alleles, even with smaller core collection sizes [24].

In this study, we systematically evaluated seven optimization strategies implemented in Core Hunter v3 (EN-MR, EN-CE, AN-CE, AN-MR, SH, HE, and multi-objective combination) under both stratified and unstratified sampling frameworks. Across all genetic diversity metrics—including PIC, He, Ho, I, and J′—stratified sampling consistently outperformed unstratified sampling. This supports Brown’s [25] hypothesis that ecological or geographical stratification can reduce redundancy and preserve subgroup-specific variation. The two-level design of “inter-group representativeness + intra-group diversity maximization” enabled dual optimization in both structural integrity and genetic coverage of the core collection.

Among all the strategies, expected heterozygosity (HE) demonstrated the most stable performance under the stratified framework, consistent with findings in wheat by Soleimani et al. [24]. At the 20% sampling intensity, the S-HE20 collection achieved the highest genetic diversity values (He = 0.366, PIC = 0.290, I = 0.530), indicating its superiority in maintaining within-locus variability and balanced allele frequencies. By contrast, S-HE10 achieved the highest observed heterozygosity (Ho = 0.516) and evenness (J′ = 0.766) at a lower sampling intensity. These preferences differ from those observed in potato by Manrique-Carpintero et al. [26], who favored AE-MR based on MR distance, suggesting that clonally propagated species like moso bamboo tend to retain intra-locus heterozygosity rather than maximizing inter-sample divergence. In moso bamboo, the predominantly clonal mode of reproduction preserves within-locus allelic variability and mitigates allele fixation, consistent with the excess heterozygosity observed across groups (Ho > He). This mechanism explains the strong performance of the S-HE20 collection in maintaining representative genetic diversity. In contrast, EN-based distance strategies prioritize maximizing divergence among sampled individuals, which can compromise population representativeness. This reflects a key trade-off in core collection development: divergence-oriented methods capture broader genetic distances, whereas HE-based approaches better retain population-level allelic balance in predominantly clonal species.

Further validation using phenotypic data confirmed the effectiveness of the S-HE20 core collection. It preserved the mean values of the full population (MD = 0) and captured 82.32% of the total phenotypic variation (CR), outperforming S-HE10 (CR = 72.82%). It also exhibited strong performance in capturing trait extremes, as shown by high variance-related indices (VD = 80.33%, VR = 101.72%). In terms of genetic diversity, S-HE20 maintained comparable diversity levels (He, PIC) to the original collection, while significantly increasing observed heterozygosity (Ho = 0.5036 vs. 0.4754), indicating improved within-locus variability. PCA and phylogenetic tree analyses further confirmed the S-HE20 core collection retained the overall population structure the of the original collection and captured marginal and unique accessions. These findings confirm its strong representativeness and broad genetic coverage, thereby providing a valuable resource for future genetic studies and breeding programs.

## Conclusions

In this study, we constructed the first core collection of moso bamboo based on whole-genome resequencing data from 432 accessions covering a broad spectrum of its distribution range. By systematically evaluating different strategies, the optimal approach was identified as stratified sampling combined with the expected heterozygosity algorithm at a 20% sampling proportion, resulting in a core collection of 84 accessions. This approach effectively reduced genetic redundancy while maximizing both genetic diversity and phenotypic representativeness. The resulting core collection provides a valuable foundation for the accurate conservation, germplasm innovation, and molecular-assisted breeding of moso bamboo.

## Methods

### Sample collection

Due to the strong growth and clonal propagation ability of moso bamboo, which spreads via rhizomes, care was taken to avoid sampling genetically identical individuals. To maximize genetic diversity and avoid redundancy, a grid-based random sampling strategy was adopted. Sampling sites were established at the intersections of a 150 km latitude–longitude grid across the whole distribution range of moso bamboo in China. At each site, 3–6 bamboo individuals were sampled, ensuring a minimum distance of 1 km between individuals. Given the extensive human influence on moso bamboo distribution and the difficulty of distinguishing between natural and cultivated stands, only populations that, according to local forestry bureaus and bamboo farmers, had been established for over 20 years were included. In total, 432 moso bamboo individuals were sampled, covering 16 distinct geographic regions and representing the full range of moso bamboo habitats in China (Fig. 3). Detailed information on the collected accessions is provided in Additional file 1: Table S1. A schematic flow diagram summarizing the full workflow—from field sampling to quality control, population structure inference, candidate core construction, and final core selection—is provided in Additional file 6: Fig. S1.

### DNA extraction and sequencing

Fresh young leaves were collected from second-year moso bamboo plants, desiccated with silica gel, and stored at − 80 °C prior to DNA extraction. Genomic DNA was isolated following the cetyltrimethylammonium bromide (CTAB) protocol [27]. DNA quality and purity were evaluated via 0.8% agarose gel electrophoresis, and concentrations were determined using a NanoDrop 2000 spectrophotometer (Thermo Fisher Scientific, USA). Paired-end sequencing was carried out on the DNBSEQ-T7 platform (MGI Tech) at an average depth of 20 × per sample.

### Variant calling and SNP Filtering

Adapter sequences and low-quality bases were trimmed from the raw sequencing reads using fqtools_plus [28]. Reads were discarded if more than 5% of the bases were “N” or if over 50% of the bases had a quality score < 20. The filtered high-quality reads were aligned to the moso bamboo reference genome [29] using BWA-MEM v0.7.12 [30] and the resulting alignments were sorted with Samtools v1.10 [31]. PCR duplicates were identified and removed using the *MarkDuplicates* module in Picard v2.2 (http://broadinstitute.github.io/picard). Variant calling for all 432 individuals was performed using the *HaplotypeCaller* tool in GATK v4.1.2 [32] with the “-ERC GVCF” option. The generated GVCF files were merged, and SNPs were jointly genotyped using *GenomicsDBImport*, *GenotypeGVCFs*, and *SelectVariants* modules in GATK v4.1.2. Raw SNPs were filtered using the following GATK hard-filtering criteria: QD < 2.0, MQ < 40.0, FS > 60.0, SOR > 3.0, MQRankSum < −12.5, and ReadPosRankSum < −8.0. To further improve data quality, the GATK-filtered SNPs were refined using bcftools v1.17 (https://www.htslib.org/doc/1.0/bcftools.html), removing loci with sequencing depth outside the acceptable range (mean ± SD, i.e., AVG(FMT/DP) < 10 or > 30) and retaining only biallelic SNPs (-m2 -M2). PLINK v1.90 [33] was then used to exclude SNPs with missing data rates > 10% and SnpEff v5.0 [34] was employed for SNP annotation. After filtering, 58,430,318 bi-allelic SNPs remained for downstream analyses. All command-line parameters and exact software settings used in this workflow are provided in the supplementary methods to ensure full reproducibility (Additional file 7: supplementary methods).

### Population structure analysis

We first employed PLINK v1.90 [33] with the parameters “indep-pairwise 100 10 0.2” to obtain a linkage disequilibrium (LD)-pruned SNP set with a minor allele frequency (MAF) > 5%, resulting in 329,747 independent SNPs for population structure analyses. A maximum-likelihood (ML) phylogenetic tree was generated using IQ-TREE [35] with the options *-m MFP -B 1000 -nt 60* and visualized in iTOL (https://itol.embl.de/). Principal component analysis (PCA) was performed in PLINK v1.90, and population structure was inferred using ADMIXTURE v1.3.0 [36] with the parameters *–cv -B 100 -j 2* for K values ranging from 1 to 16. The resulting structure plots were visualized using the “pophelper” package in R (https://github.com/royfrancis/pophelper).

### Genetic diversity analysis

To assess genetic diversity among the moso bamboo accessions, we calculated multiple indices, including observed heterozygosity (Ho), expected heterozygosity (He), polymorphic information content (PIC), Shannon’s diversity index (I), and genetic evenness (J′). The indices Ho, He, and PIC were computed using the R package “snpReady” (https://github.com/italo-granato/snpReady) [37], whereas I and J′ were calculated following the methods of Hennink [38] and Pielou [39], respectively.

### Establishment of the core collection

For the construction of the core collections, two sampling strategies were employed: unstratified sampling, in which all accessions were treated as a single group, and stratified sampling, in which accessions were grouped based on genetic structure and phylogenetic relationships, and samples were proportionally drawn from each group. A total of seven sampling algorithms were tested under five sampling intensities (10%, 15%, 20%, 25%, and 30%) using the R package Core Hunter v3 (https://github.com/corehunter/corehunter3) [19]. The algorithms are described as follows:

1. EN-MR (Entry-to-Nearest-Entry with Modified Rogers’ Distance): Aims to maximize the mean genetic distance from each selected accession and its closest neighbor within the core collection, based on Modified Rogers’ Distance (MRD). This strategy reduces redundancy and enhances intra-core diversity. MRD is sensitive to both common and rare alleles, making it suitable for detecting fine-scale genetic differentiation [19, 40].
2. EN-CE (Entry-to-Nearest-Entry with Cavalli–Sforza and Edwards’ distance): Similar to EN-MR, this method also maximizes the pairwise distance within the core collection, but uses Cavalli–Sforza and Edwards’ distance (CSE), which emphasizes allele frequency differentiation and is less influenced by rare alleles, thereby reflecting broader diversity trends.
3. AN-CE (Accession-to-Nearest-Entry with Cavalli–Sforza and Edwards’ distance): Minimizes the mean distance from each accession in the entire dataset to its closest representative in the core collection, using the CSE metric. This method emphasizes representativeness by ensuring that the core broadly captures the full genetic landscape [19, 40].
4. AN-MR (Accession-to-Nearest-Entry with Modified Rogers’ Distance): A variant of AN-CE that uses MRD as the distance metric. It prioritizes representativeness while placing more emphasis on capturing subtle allelic variations, including low-frequency alleles.
5. SH (Shannon’s diversity index): Selects accessions to maximize overall allelic richness by weighting rare alleles more heavily. This index increases when alleles are evenly distributed and uniquely represented, making it effective in preserving low-frequency alleles and maximizing diversity breadth [23, 41].
6. HE (Expected heterozygosity): Aims to maximize the expected heterozygosity across loci, reflecting within-locus genetic variability. HE gives equal weight to all loci and favors collections that retain heterozygosity and avoid allele fixation, thus promoting overall genetic variability [21, 42].
7. Multi-objective optimization: Combines EN-MR and SH objectives with equal weighting (0.5), balancing pairwise genetic dissimilarity and allelic richness to obtain core collections with both structural and allelic diversity.

In total, 70 candidate core collections were generated (two grouping strategies × seven sampling methods × five sampling intensities). All subsets were constructed using the sampleCore() function with specified objective parameters and were subsequently evaluated for their genetic and phenotypic diversity.

### Evaluation of the core collections

To evaluate the representativeness of the candidate core collections constructed under different strategies, a two-step evaluation framework was applied. In the first stage, genetic diversity (Ho, He, PIC, I) and genetic evenness (J′) of all 70 candidate cores was evaluated. Candidate core collections with relatively high diversity were retained. In the second stage, the selected core collections were further evaluated for phenotypic representativeness using 15 traits: diameter at breast height (DBH), basal diameter (BD), total culm height (TCH), total number of nodes (TNN), height to the first branch (HFB), number of nodes below the first branch (NNFB), internode length (IL), crown width (CW), average culm base wall thickness (ACWTB), average wall thickness at breast height (AWTH), culm cavity diameter (CCD), leaf thickness (LT), leaf area (LA), leaf length (LL), and leaf width (LW). These traits were chosen for their strong association with genetic variation, their relevance to growth, morphology, and functional characteristics of moso bamboo, and their importance to its economic value. Phenotypic evaluation involved four statistical indicators: Mean Difference Percentage (MD) = (*S*_*t*_/n) × 100%, where *St* represents the number of traits with significant differences (α = 0.05) in t-tests between the core and original collections, and *n* denotes the total number of traits. Variance Difference Percentage (VD) = (*S*_*F*_*/* n) × 100%, where *SF* is the number of traits with significant differences (α = 0.05) in F-tests between the core and original collections. Coincidence Rate of Range (CR) = $\frac{1}{n}\sum_{i=1}^{n}\frac{{R}_{c(i)}}{{R}_{I(i)}}$ × 100%, where *Rc(i)* and *RI(i)* are the range of the *i*-th trait in the core and original collections, respectively. Coefficient of Variation Change (VR) = $\frac{1}{n}\sum_{i=1}^{n}\frac{{CV}_{c(i)}}{{CV}_{I(i)}}$ × 100%, where *CV*_*c(i)*_ and *CV*_*I(i)*_ are the coefficient of variation for the i-th trait in the core and original collections, respectively [43]. An effective core collection should meet the criteria of MD < 20% and CR > 80%. In addition, a higher CR, VD, and VR, along with a lower MD, indicate a stronger representative quality of the core collection [43]. Based on this two-step evaluation framework integrating genetic and phenotypic data, the optimal core collections were identified for subsequent validation analysis.

### Validation of core collections

To verify the quality of the optimal core collections, we performed t-tests to compare it with the original collection in terms of genetic diversity indices (Ho, He, PIC, I), genetic evenness (J′) and 15 phenotypic traits. For each phenotypic trait, we assessed differences in maximum value, minimum value, coefficient of variation (CV), and mean. Principal component analyses (PCA) on genotypic and phenotypic datasets were carried out to examine the structural composition of the core collection.

## Supplementary Information

Supplementary Material 1.

Supplementary Material 2.

Supplementary Material 3.

Supplementary Material 4.

Supplementary Material 5.

Supplementary Material 6.

Supplementary Material 7.

## Data Availability

All sequencing data for 432 individuals in this study have been deposited in the National Genomics Data Center (https://ngdc.cncb.ac.cn) under accession number PRJCA046029.

## Abbreviations

Ho: Observed heterozygosity
He: Expected heterozygosity
PIC: Polymorphism information content
I: Shannon’s diversity index
J′: Genetic evenness
GATK: Genome Analysis Toolkit
MAF: Minor allele frequency
PCA: Principal component analysis
SNP: Single-nucleotide polymorphism
LD: Linkage disequilibrium
MD: Mean difference percentage
CR: Coincidence rate of range
VD: Variance difference percentage
VR: Coefficient of variation change
DBH: Diameter at breast height
BD: Basal diameter
TCH: Total culm height
TNN: Total number of nodes
HFB: Height to the first branch
NNFB: Number of nodes below the first branch
IL: Internode length
CW: Crown width
ACWTB: Average culm base wall thickness
AWTH: Average wall thickness at breast height
CCD: Culm cavity diameter
LT: Leaf thickness
LA: Leaf area
LL: Leaf length
LW: Leaf width
ML: Maximum likelihood
EN-MR: Entry-to-Nearest-Entry with Modified Rogers’ Distance
EN-CE: Entry-to-Nearest-Entry with Cavalli–Sforza and Edwards’ distance
AN-CE: Accession-to-Nearest-Entry with Cavalli–Sforza and Edwards’ distance
AN-MR: Accession-to-Nearest-Entry with Modified Rogers’ Distance
MRD: Modified Rogers’ Distance
CSE: Cavalli–Sforza and Edwards’ distance
