# Supplementary figures and images for: Cutting through the clones: genomic strategies for core collection development in moso bamboo
Source: BMC Genomics. 2026 Jan 20;27:97. doi: 10.1186/s12864-026-12548-7 (PMC12837288; doi:10.1186/s12864-026-12548-7)

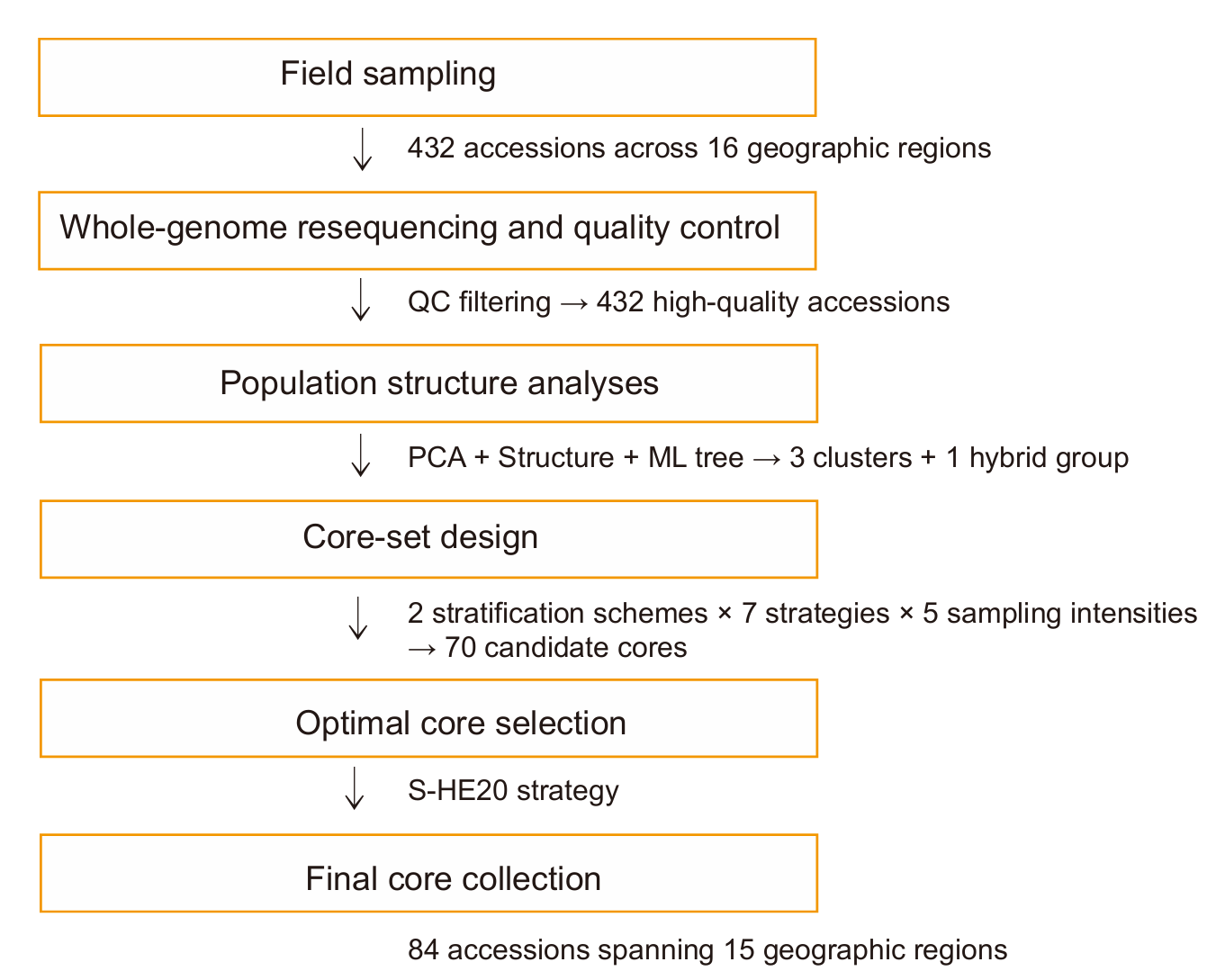


Fig. S1. Workflow of core collection development from field sampling to final core selection

Supplement: Supplementary file 6 — Supplementary Material 6. [file 12864_2026_12548_MOESM6_ESM.docx]
