# Supplementary material for: Cutting through the clones: genomic strategies for core collection development in moso bamboo
Source: BMC Genomics. 2026 Jan 20;27:97. doi: 10.1186/s12864-026-12548-7 (PMC12837288; doi:10.1186/s12864-026-12548-7)
Supplement: Supplementary file 7 — Supplementary Material 7. [file 12864_2026_12548_MOESM7_ESM.docx]

Supplementary Methods

**1. fqtools**

fqtools_plus filter --m 4 --ql 19 --Q 0.5 --n 0.05 --polyN G --tailen 75 --adapt1 GATCGGAAGAGCACACGTCTGAACTCCAGTCAC --adapt2 AATGATACGGCGACCACCGAGATCTACAC input_R1.fq.gz input_R2.fq.gz output_R1.clean.fq.gz output_R2.clean.fq.gz

**2.BWA-MEM v0.7.12**

bwa mem -t 10 -R '@RG\tID:$sp\tSM:$sp\tPL:illumina' $ref $fq1 $fq2 2>$sp.bwa.log

**3.Samtools v1.10**

samtools sort -@ 10 -m 30G -o $sp.sort.bam –

**4.Picard v2.2**

java -Xmx30g -XX:ParallelGCThreads=10 -jar /opt/picard.jar MarkDuplicates I=$sp.sort.bam O=$sp.sort.markdup.bam CREATE_INDEX=true REMOVE_DUPLICATES=true M=$sp.marked_dup_metrics.txt

**5.GATK v4.1.2**

***HaplotypeCaller module***

--java-options \"-Xmx30g -Djava.io.tmpdir=./tmp\" HaplotypeCaller -R $ref -I $bam -L $chr -ERC GVCF -O $sample/$sample.$chr.g.vcf.gz 1>$sample/$sample.$chr.HC.log 2>&1

***GenomicsDBImport module***

--java-options \"-Xmx50g -Djava.io.tmpdir=./tmp -DGATK_STACKTRACE_ON_USER_EXCEPTION=true\" GenomicsDBImport --sample-name-map gvcf.$chr.map --genomicsdb-workspace-path genomeDB.$chr -L $chr --reader-threads 2 --batch-size 100 --tmp-dir ./tmp 1>$chr.GenomicsDBImport.log 2>&1"

***GenotypeGVCFs module***

--java-options \"-Xmx80g -Djava.io.tmpdir=./tmp\" GenotypeGVCFs -R $ref -V gendb://genomeDB.$chr -O $chr.raw.vcf.gz 1>$chr.GenotypeGVCFs.log 2>&1 "

***SelectVariants module***

SelectVariants -R ../01.ref/genome.fasta -V all.merge_raw.vcf --select-type SNP -O all.raw.snp.vcf

***Variant filtering module***

--java-options "-Xmx30g -Djava.io.tmpdir=./tmp" VariantFiltration -R ../01.ref/genome.fasta -V all.raw.snp.vcf --filter-expression "QD < 2.0 || MQ < 40.0 || FS > 60.0 || SOR > 3.0 || MQRankSum < -12.5 || ReadPosRankSum < -8.0" --filter-name 'SNP_filter' -O all.filter.snp.vcf

SelectVariants -R ../01.ref/genome.fasta -V all.filter.snp.vcf --exclude-filtered -O all.filtered.snp.vcf

**6.bcftools v1.17**

bcftools view -Oz -o all.biallelic.vcf.gz --threads 2 -m2 -M2 -v snps all.filter.snp.vcf.gz

**7.PLINK v1.90**

***MAF filter***

plink --vcf all.biallelic.filtered.tagged.vcf --geno 0.1 --maf 0.05 --biallelic-only strict --out all.DPmissingmaf --recode vcf-iid --allow-extra-chr --set-missing-var-ids @:# --keep-allele-order

***LD filter***

plink --vcf all.DPmissingmaf.vcf --indep-pairwise 100 10 0.2 --out tmp.ld --allow-extra-chr --set-missing-var-ids @:#

plink --vcf all.missing_maf.vcf --make-bed --extract tmp.ld.prune.in --out all.LDfilter --recode vcf-iid --keep-allele-order --allow-extra-chr --set-missing-var-ids @:#

**8.ADMIXTURE v1.3.0**

admixture --cv -B100 -j2 chrunrelate.bed $k 1>admix.2.log 2>&1

**9.IQ-TREE v2**

iqtree2 -s all.DPMISSMAF5LD.phy -st DNA -nt 30 -m MFP -redo -B 1000 -bnni --prefix iqtree 2>iqtreeGTR.run1.log

**10.Core Hunter v3**

core_EN_MR <- sampleCore(my.data, size = sample_size, obj = objective("EN", "MR"))

core_EN_CE <- sampleCore(my.data, size = sample_size, obj = objective("EN", "CE"))

core_AN_CE <- sampleCore(my.data, size = sample_size,obj = objective("AN", "CE"))

core_AN_MR <- sampleCore(my.data, size = sample_size, obj = objective("AN", "MR"))

core_Shannon <- sampleCore(my.data, size = sample_size,obj = objective("SH"))

core_He <- sampleCore(my.data, size = sample_size, obj = objective("HE"))

core_multi <- sampleCore(my.data, size = sample_size, obj = list( objective("EN", "MR", weight = 0.5), objective("SH", weight = 0.5)))
